# Current state of national TB laboratory networks in Europe: achievements and challenges

**DOI:** 10.5588/ijtld.21.0520

**Published:** 2022-01-01

**Authors:** K. Klaos, Y. Holicka, R. Groenheit, C. Ködmön, M. J. Van der Werf, V. Nikolayevskyy

**Affiliations:** 1 Department of Mycobacteriology, United Laboratories, Tartu University Hospital, Tartu, Estonia; 2 Department of Pulmonary Medicine, University of Tartu, Tartu, Estonia; 3 Public Health England, London, UK; 4 Public Health Agency of Sweden, Stockholm, Sweden; 5 European Centre for Disease Prevention and Control, Solna, Sweden; 6 Department of Infectious Diseases and Immunity, Imperial College, London, UK

Dear Editor,

TB has caused human suffering for centuries.^1^ During the long history of co-existing with *Mycobacterium tuberculosis*, mankind has implemented techniques to fight the pathogen. Nevertheless, TB disease persists (despite bloodletting, the “royal touch”, sanatoriums, induced pneumothorax or the use of antibiotics) and still causes 1.4 million deaths annually.^2^,^3^ To tackle a surge in TB incidence after the Civil War in the former Union of Soviet Socialist Republics (USSR), a network of TB dispensaries and sanatoria was established before World War II to provide harmonised services for TB diagnosis and treatment.^4^ Similar centrally coordinated TB services were established in other European countries (e.g., the German Democratic Republic and Italy).^5^,^6^ However, after the collapse of the USSR, declining living standards led to interruptions in drug supply and a breakdown of the TB prevention and care system. This has contributed to an increase in TB incidence and spread of multidrug-resistant TB (MDR-TB) in countries such as Estonia, Latvia, Lithuania, Belarus, Bosnia and Herzegovina, Moldova, Romania and Armenia.^7^–^9^ At the same time, economic progress and higher living standards led to a decline in TB incidence in many other western European countries.^9^,^10^ In order to cope with the extensive spread of TB, many Eastern and Central European states established local TB programmes adopting hybrid practices from Soviet dispensaries and from Western European countries, creating in-country tiered laboratory networks. This was in contrast to other European Union (EU) countries with largely decentralised TB clinical and laboratory systems.

The first European Reference Laboratory Network for TB (ERLN-TB) co-ordinated by the European Centre for Disease Prevention and Control (ECDC) was established in 2009 and included all EU and European Economic Area (EEA) countries. The network aimed to provide technical support, methods harmonisation, and training and cooperation opportunities.^11^,^12^ In 2015, a survey conducted within the network revealed significant differences in structure and functionality of national TB laboratory networks in EU and EEA countries (unpublished data). The aim of the current study was to further analyse the role and functionality of TB laboratory networks in the EU/EEA countries and explore ways of supporting these networks. To this end, the ECDC, together with a working group within the second European Reference Laboratory Network for TB (ERLTB-Net-2), conducted a self-assessment survey. The survey questionnaire included questions about the appointment and the role of the National Reference Laboratory (NRL), the maintenance and structure of the TB laboratory network, the availability of human, biosafety, and financial resources, as well as the main strengths and weaknesses, and prioritised needs. All ERLTB-Net-2 members were invited to participate in the survey through an online platform (Survey Generator^TM^; Alstra, Stockholm, Sweden) and gave their consent to analyse the data. The aim of this article was to aggregate findings from the survey and to highlight and discuss relevant results.

All 30 invited NRLs (100%) completed the questionnaire in February 2020. The combined results showed that the Ministry of Health or the national Health Board selects and appoints the NRL for TB in 80% of the participating countries. In three countries (10%), NRLs are selected at regular intervals on a competitive basis by an independent committee. To note, despite many NRLs being appointed by a national competent body, there are no legal regulations or guidance for the operation of the NRL or the national TB laboratory network in 70% of the countries, and no defined national standards for the services of the NRL or the national TB laboratory network in 60% of these countries. Furthermore, there is no financial support from government for diagnostic reference services in six countries (20%), and seven (23%) reported no financial support for reference services (e.g., provision of training or quality assessment). Seven participants stated that there is no national TB laboratory network in their country; in five of these countries the NRL is the only laboratory performing TB diagnosis. Seven countries (23%) have an official national TB laboratory network and respondents of five of these countries consider their official network to be functioning well (see Figure). Unofficial national TB laboratory networks exist in 16 (53%) countries. According to the respondents, the functioning of unofficial networks depends on initiatives taken by the NRL, and is mainly based on goodwill, pro-active communication and historical links, but not on national guidelines or regulations. Nine of the 16 NRLs that had established an unofficial TB laboratory network considered it to be functioning well. The unregulated voluntary status of an unofficial laboratory network may result in a lack of knowledge in the NRL about the methods used in peripheral laboratories and the proficiency level for these methods. In addition, implementation of novel diagnostic methods may be challenging due to the lack of a central authority and support. A challenge for the many of the NRLs within a TB laboratory network is that they do not play an authoritative role, that is, they can only give advice, suggestions and technical support to peripheral laboratories showing suboptimal performance; this challenge was reported by 21 of the 23 NRLs maintaining a TB laboratory network. Six (26%) out of 23 countries with an existing official or unofficial TB laboratory network listed the presence of too many laboratories performing TB diagnostics to be a key weakness of their laboratory network, along with lack of human resources and underfunding, although our results indicate that financing has improved in recent years.^13^ With steadily declining TB incidence^14^ and increasing availability of rapid molecular tests, samples processed using traditional diagnostic methods are too few for many laboratories to maintain their proficiency. Furthermore, many laboratories testing relatively few samples may not be cost-effective. Five of the participating NRLs did not know how many laboratories performed TB laboratory diagnostics tests in their own country. Furthermore, five other respondents stated that it is difficult to categorise laboratories into the WHO Global Laboratory Initiative TB laboratory levels because different categories are used in their country.^15^ Of note is the variation in the number of new and relapse cases diagnosed per laboratory among the EU/EEA countries, with a median of 42 per year (interquartile range 27–64; min 7, max 1350; 2019 data).^3^ Six respondents stated that they currently do not have a biosafety level 3 (BSL-3) facility in their NRL; however, two of these laboratories meet the criteria for BSL-3 with the exception of the requirement for negative pressure environment.

**Figure:**
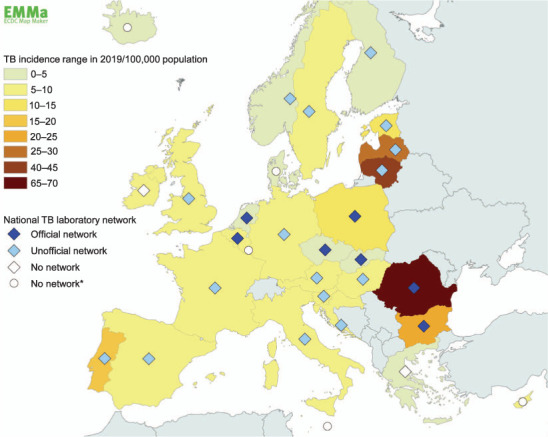
Map, displaying TB incidence ranges for 2019 (WHO, 2020)^3^ and the state (official/unofficial or no network) of national TB laboratory networks in European Union/European Economic Area countries, constructed using the ECDC Map Maker tool. *Only one TB diagnostic laboratory in the country. ECDC = European Centre for Disease Prevention and Control.

Our survey shows that TB laboratory networks exist widely in the EU/EEA, in both official (in 23% of countries) and unofficial (in 53% of countries) form. These networks provide connections between different laboratory levels to support fast, high-quality and cost-effective services for diagnosing TB. There was no indication that countries with a relatively high TB incidence had more laboratories or an official or unofficial national TB laboratory network (Figure). The weaknesses highlighted in this study highlight the need for legal and infrastructural changes, limiting the number of TB laboratories, where applicable, and restructuring TB diagnostic services. Our results show that problems with infrastructure, the number of laboratories and funding of reference services persist despite improvements in laboratory financing. Targeted support and training, technical assistance or task force visits to EU/EEA countries to further develop their TB laboratory networks, remains an important activity for the ERLTB-Net-2 network.
